# A Digital PCR Assay to Quantify the Percentages of Hulled vs. Hulless Wheat in Flours and Flour-Based Products

**DOI:** 10.3390/biology10111138

**Published:** 2021-11-05

**Authors:** Caterina Morcia, Raffaella Bergami, Sonia Scaramagli, Chiara Delogu, Lorella Andreani, Paola Carnevali, Giorgio Tumino, Roberta Ghizzoni, Valeria Terzi

**Affiliations:** 1 Consiglio Per la Ricerca in Agricoltura e L’analisi Dell’economia Agraria-Centro di Ricerca Genomica e Bioinformatica (CREA-GB), Via San Protaso 302, 29017 Fiorenzuola d’Arda, PC, Italy; caterina.morcia@crea.gov.it (C.M.); roberta.ghizzoni@crea.gov.it (R.G.); 2 Coop Italia, Via del Lavoro, 6/8, I-40033 Casalecchio di Reno, BO, Italy; raffaella.bergami@coopitalia.coop.it (R.B.); sonia.scaramagli@coopitalia.coop.it (S.S.); 3 Consiglio Per la Ricerca in Agricoltura e L’analisi Dell’economia Agraria-Centro di Ricerca Difesa e Certificazione (CREA-DC), Via Emilia km 307, 26838 Tavazzano, LO, Italy; chiara.delogu@crea.gov.it (C.D.); lorella.andreani@crea.gov.it (L.A.); 4 Barilla S.p.A., Via Mantova 166, I-43122 Parma, PR, Italy; paola.carnevali@barilla.com; 5 Plant Breeding, Wageningen Plant Research, Wageningen University & Research, Droevendaalsesteeg 1, 6708 PB Wageningen, The Netherlands; giorgio.tumino@wur.nl

**Keywords:** hulled wheats, einkorn, emmer, spelt, *Triticum monococcum*, *Triticum dicoccum*, *Triticum spelta*, authenticity, quantification, dPCR

## Abstract

**Simple Summary:**

The agri-food market is currently showing interest in hulled wheat-based products, in particular emmer and spelt. These wheats were rediscovered as ingredients for both traditional and innovative food products. Since hulled wheats’ commodity value is higher than common and durum wheat, it is useful to have an analytical system that allows to control and quantify the actual presence of einkorn, emmer and spelt and, therefore, check the authenticity of derived products. With this aim, we developed an analytical assay based on digitalPCR, which has been able to discriminate between hulled (i.e., einkorn, emmer and spelt) and common or durum wheats and to give a quantification. The assay can be used along production chains, from raw materials to final food products.

**Abstract:**

Several food products, made from hulled wheats, are now offered by the market, ranging from grains and pasta to flour and bakery products. The possibility of verifying the authenticity of wheat species used at any point in the production chain is relevant, in defense of both producers and consumers. A chip digital PCR assay has been developed to detect and quantify percentages of hulless (i.e., common and durum wheat) and hulled (i.e., einkorn, emmer and spelt) wheats in grains, flours and food products. The assay has been designed on a polymorphism in the miRNA172 target site of the AP2-5 transcription factor localized on chromosome 5A and involved in wheat spike morphogenesis and grain threshability. The assay has been evaluated even in a real-time PCR system to assess its applicability and to compare the analytical costs between dPCR and real-time PCR approaches.

## 1. Introduction

The ethnobotanical Italian term “farro” indicates the complex of einkorn, emmer and spelt, the earliest wheats to be cultivated, and therefore, identified as “ancient grains”. Ancient wheats are characterized by three different levels of ploidy: einkorn (*T. monococcum* L.) is diploid (AA; 2*n* = 2*x* = 14), emmer (*T. turgidum* L. spp. *dicoccum* Schrank ex Schübler) is tetraploid (AABB; 2*n* = 4*x* = 28), whereas spelt (*T*. *aestivum* subsp. *spelta*) is hexaploid (AABBDD; 2*n* = 6*x* = 42). These plants, primarily domesticated in the Fertile Crescent area where their wild ancestors are still present [1], are among the founder crops of agriculture [2].

Einkorn is today present in isolated mountain areas of a few Mediterranean and European countries only [3] and can be considered a relic crop.

Emmer, domesticated 10,000 years ago from *Triticum dicoccoides* [4] represents today about 1% of the total world wheat area. It is cultivated as a minor crop in Iran, Eastern Turkey, Transcaucasia, the Volga Basin, ex-Yugoslavia, Central Europe, Italy and Spain [5], even though it remains an important plant in India, Ethiopia and Yemen [6]. In Europe, spelt became cultivated as far back as 7000–8000 BCE in the Neolithic period [7] and became the most important cereal in Northern and Central Europe starting from the Bronze Age. Today, spelt cultivation is mainly restricted to marginal areas in eastern Europe, Germany, Belgium, Austria, Switzerland, Slovenia, the Asturias region of Spain and Italy [8].

Ancient hulled wheats, widely cultivated in the past, were, starting from 19th century, replaced by naked wheats; however, in the last years, a trend reversal supported by consumers’ interest in traditional crops and derived food, nutritional peculiarities of hulled wheats and their aptitude to organic farming has been observed [9,10,11]. As a result of this market trend, an increase in cultivation area has been observed in several countries, including Italy.

In Italy, the hulled wheat that is typically used is emmer and the most important area of its cultivation, estimated around 4000 ha, is Central/Southern Italy. Within this area, specific ecotypes have been fixed by long time in situ reproduction. Such landraces, therefore, are typical of their own cultivation area. This cultivated area hosts emmer varieties both selected among landraces and modern cultivars, obtained by crossing cultivated emmer and durum wheat [12].

Several food products, made from hulled wheats, are now offered by the market, ranging from grains and pasta to flour and bakery products. Farro price on the Italian cereal market is significantly higher in comparison with naked wheats, ranging from 30% higher than common wheat to 15% higher than durum wheat [13]. This significant difference in commodity value, due to the easier handling and processing of naked wheats, can be the motives for alimentary frauds based on farro replacement with common or durum wheats. This implies that the possibility of verifying the authenticity of the wheat species used at any point in the production chain is relevant, in defense of both producers and consumers.

Some analytical procedures, reported in Table 1, have been proposed to track hulled wheats.

Most of the proposed assays are DNA-based methods used for the identification and quantification of spelt. Moreover, assays developed by Voorhuijzen et al. [17] and by Foschi et al. [21] are focused on the traceability of accessions specifically cultivated in Italian environments. Tubulin-based DNA barcode, multiple gene targets, *γ-gliadin* polymorphisms and *Q-locus* polymorphisms have been exploited using different technologies, ranging from PCR to microarray, up to the very recent digitalPCR [22].

The tubulin-based polymorphism (TBP) profiling developed by Silletti et al. [19] has the peculiarity to be the only DNA-based untargeted method, not requiring any prior genome sequence information and able to profile any plant species with universal primer pairs. The authors suggest that this method is a useful first screening step, which can be complemented by target quantitative analysis, performed by qPCR or other methods.

*γ-gliadin* polymorphisms were exploited in the analytical protocols developed by Mayer et al. [16] and by Curzon et al. [20].

Mayer et al. [16] proposed two alternative methods for the detection and quantification of spelt flour “adulteration” with soft wheat: a restriction fragment length (RFLP) analysis, combined with lab-on-a-chip capillary gel electrophoresis (LOC-CE) for the simple detection and a real-time PCR for the quantification of soft wheat “adulterations” in spelt.

The study of Curzon et al. [20] has the same objective, i.e., the identification of common wheat adulteration in spelt. In this study, markers for *γ-gliadin-D*, *γ-gliadin-B* and the *Q*-gene were used, alongside a phenotypic assessment based on near-infrared spectroscopy (NIRS). The *γ-gliadin* markers demonstrated low diagnostic power in comparison to the *Q*-gene marker and to the NIR predictions.

Asakura et al. [15] developed a method based on polymerase chain reaction-restriction fragment length polymorphism (PCR-RFLP) to distinguish between the Q and q alleles. PCR-RFLP analysis was extended to six conserved single nucleotide polymorphisms in common wheat and wild and cultivated einkorn, emmer and timopheevi wheat.

Q locus polymorphism was exploited even by Voorhuijzen et al. [17] to develop a DNA-based multiplex detection tool based on padlock probe ligation and microarray detection (PPLMD) for the detection of (un-)intentional adulteration of Farro della Garfagnana with different species. This approach, developed on grain samples, is sensitive enough to track the presence of 5% contaminant plant species, and therefore, it can be applied to check the purity of a premium food such as Farro della Garfagnana grains.

The recent study of Köppel et al. [22] describes a duplex droplet digital PCR (ddPCR) for the detection and quantification of contaminations by common wheat in food products made from spelt. The authors take into account both a single nucleotide polymorphism (SNP) in the Q locus, as well as a short sequence of the γ-gliadin gene. The SNP in the Q locus was able to discriminate all the tested spelt cultivars from common wheat cultivars.

The choice, made by several authors, of the Q locus as a useful analytical target to discriminate between free- and not-free threshing wheats is the logical consequence of the historical use of key morphological descriptors. Common and durum wheat kernels are naked, whereas einkorn, emmer and spelt are hulled. The naked/hulled seed trait is, therefore, one of the major morphological characters to discriminate the common and durum wheat grains from einkorn, emmer and spelt grains. This trait is genetically determined, not influenced by the cultivation environment, and with a key role in the wheat domestication process. Several studies have contributed to highlight the genetics of the trait. Pioneering in this regard was the work of Nilsson-Ehle [23], in which for the first time the Q-locus was identified as involved in wheat spike morphology and pleiotropically, affecting many other agronomic traits, such as free-threshing. Q-locus was assigned to the long arm of chromosome 5A and its molecular cloning showed that it belongs to the APETALA2 transcription factors [24]. Q-locus, similar to other AP2-like genes, has a miR172 target site within the coding region that can modulate the mRNA stability and can have an impact on several developmental processes in several species, including maize [25]. In wheat, Debernardi et al. [26] demonstrated the miR172 key role in spike morphogenesis and a sequence variation at the miR172 target site between Q and q alleles involved in the grain threshability trait.

Such polymorphism has been exploited in our study and a new digitalPCR assay has been developed that can identify and quantify all kind of hulled wheats, i.e., einkorn, emmer and spelt, and can distinguish them from naked wheats in both raw materials and processed products.

## 2. Materials and Methods

### 2.1. Samples

The wheat genotypes reported in Table 2 have been used across the study. The hulled wheat accessions selected are all present in the Italian National Register of Variety. Moreover, two durum wheat varieties (Aureo and Iride) and two common wheat varieties (Bologna and Palesio) have been selected because they are widely cultivated in Italian environments. Durum wheat Cappelli and common wheat Apulia have been selected as representative of Italian traditional varieties.

Moreover, seeds of barley (cv Fibar), oat (cv Buffalo) and rice (cv Vialone nano), included in the CREA-GB germplasm collection, have been used to evaluate the species-specificity of the assay.

A panel of different foods, labeled as containing hulled wheats and commercially available in Italy, were bought on the market. Flour samples containing different percentages of hulled and naked wheats were produced by weighing the wheat flours and homogenizing them for 10 min. Moreover, mixed flour samples containing hulled and hulless wheats and barley have been prepared using the same approach.

### 2.2. Methods

#### 2.2.1. DNA Extraction

The seeds were milled using a Cyclotec (FOSS Italia S.r.l., Padova, Italy) at a grid diameter of 0.2 mm, avoiding any contamination between samples. DNA was extracted from three biological replicates of milled seeds using the DNeasy mericon Food Kit (Qiagen, Milan, Italy), according to manufacturer’s instructions. The evaluation of quality and quantity of the extracted DNA was performed using a Qubit™ fluorometer in combination with the Qubit™ dsDNA BR assay kit (Invitrogen by Thermo Fisher Scientific, Monza, Italy).

The same procedure was applied for the DNA extraction from flour and food samples, starting from 2 g. The evaluation of quality and quantity of extracted DNA was performed as described above.

#### 2.2.2. Chip Digital PCR

Primers and MGB probes were designed on the C/T mutation that lies within the miR172 target site in exon 10 of the AP2-5 transcription factor [23]. The Custom TaqMan^®^ SNP Genotyping assay procedure (Thermo Fisher Scientific, Monza, Italy) was used to design the allelic discrimination assay, and primers and probes are available as assay ID ANH6NUZ, Catalog n. 4332077 (Thermo Fisher Scientific, Monza, Italy). In the dPCR assay developed, the recessive allele carrying cytosine was marked with VIC, whereas the dominant allele, carrying timyne, was marked with FAM. Chip digital PCR was performed using the QuantStudioTM 3D Digital PCR System (Applied Biosystems by Life Technologies, Monza, Italy). The reaction mixture was prepared in a final volume of 16 µL consisting of 8 µL QuantStudioTM 3D Digital PCR 2X Master Mix, 0.4 µL of Custom TaqMan^®^ SNP Genotyping assay 40X (Catalogue number 4332077, Applied Biosystems by Life Technologies, Monza, Italy) containing primer and VIC/FAM-MGB probes, 1 µL of DNA (10 ng/µL) and nuclease-free water. In addition, a negative control with nuclease-free water as a template was added. A total volume of 15 µL of reaction mixture was loaded onto the QuantStudioTM 3D Digital PCR chips using the QuantStudioTM 3D Digital chip loader, according to the manufacturer’s protocol. Amplifications were performed in a ProFlexTM 2Xflat PCR System Thermocycler (Applied Biosystems by Life Technologies, Monza, Italy) under the following conditions: 96 °C for 10 min, 47 cycles of 60 °C annealing for 2 min, and 98 °C denaturation for 30 s, followed by 60 °C for 2 min and 10 °C. The fluorescent signals were detected at the end of the amplification, in an end-point mode [28]. The fluorescence data were collected in a QuantStudioTM 3D Digital PCR Instrument, and the files generated were analyzed using cloud-based platform QuantStudioTM 3D AnalysisSuite dPCR software, version 3.1.6. Each sample was analyzed in triplicate.

All the commercial samples were analyzed by the two laboratories of CREA and of Coop Italia.

#### 2.2.3. Hulless Wheat Percentage Calculation

**Figure 1:**
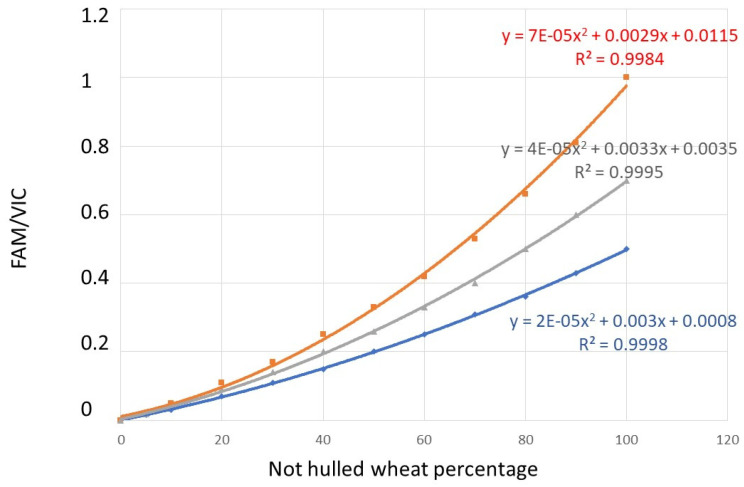
Polynomial curves reporting the theoretical relationships among FAM/VIC ratios and hulless/hulled wheats. The red curve refers to the case of durum wheat mixed with hulled wheats of any ploidy level; the blue curve refers to the case of common wheat mixed with hulled wheats of any ploidy level; the grey curve refers to a “mean situation” in which a 50:50 mixture of durum and common wheat is mixed with hulled wheats of any ploidy level.

The polynomial curves reported in Figure 1 were developed and used for hulless wheat percentage calculations. The theoretical curves were built starting from the above-listed premises and from the genetic information reported in Table 3:

1. The C allele, marked with VIC, is present in miRNA172 target site of the AP2-5 transcription factor localized on chromosome 5A in hulled wheats;
2. The T allele, marked with FAM, is present in miRNA172 target site of the AP2-5 transcription factor localized on chromosome 5A in hulless wheats;
3. The C allele is present in miRNA172 target site homoeologous regions of chromosome 5B and 5D in all wheats.

The curve was, therefore, developed considering the fact that all hulled wheats, regardless of their ploidy level, will only give a VIC signal, since the three genomes A, B and D all carry the allele with C base. On the contrary, both the durum and common wheat gave a double signal, both VIC and FAM, as both carry the T allele in genome A and the C allele in genomes B and D. In the case of monospecies hulled samples, it was, therefore, easy to highlight the exclusive presence of hulled wheats because only the VIC signal is present. In the case of monospecies samples of durum wheat, a double VIC and FAM signal of equal intensity was observed, and the FAM/VIC ratio will be equal to 1. In the case of common wheat, the VIC signal was double the FAM signal, as genome A carries the T allele, while the other two genomes B and D carry the C allele. Starting from these premises, the polynomial curve was constructed by calculating the theoretical ratios FAM/VIC in the case in which hulled and hulless are mixed. Therefore, the polynomial curve has been constructed taking into account 100% hulled wheat samples, 100% common wheat sample, 100% common wheat sample and mixed hulled/hulless samples. In Figure 1, the red curve was drawn using 100% einkorn or emmer or spelt samples, 100% durum wheat sample and samples of hulled/durum wheat in mixed percentages (between 100% hulled and 100% hulless) in 10% increments. The same approach was used to draw the blue curve, using 100% einkorn or emmer or spelt samples, 100% common wheat sample and mixed samples of hulled/common wheat between 100% hulled and 100% hulless in 10% increments. The grey curve was drawn using the 100% hulled sample, 100% hulless sample (made of 50% durum and 50% common wheat) and mixed samples of hulled/hulless wheats between 100% hulled and 100% hulless in 10% increments.

#### 2.2.4. Real-Time PCR

The same primers/probes of the digitalPCR assay were used in real-time PCR analysis. The reaction mixture was prepared in a final volume of 20 µL consisting of 10 µL of Master Mix, 0.5 µL of Custom TaqMan^®^ SNP Genotyping assay 40X (Catalogue number 4332077, Applied Biosystems by Life Technologies, Monza, Italy), 5 µL DNA template diluted at 20 ng/µL) and 4.5 µL of water. Three technical real-time PCR replicates were done for each sample and control. The PCR mixture was heated at 50 °C for 2 min and activated at 95 °C for 10 min. Forty amplification cycles were carried out at 95 °C for 15 s followed by 60 °C for 1 min. The signal detection was performed at each cycle, in real-time mode [28]. The percentage of hulled/hulless wheat was calculated as the ratio of the copy number of the hulled target gene sequence to the copy number of the target hulless gene sequence.

Different dilutions of standard samples of known hulled/hulless concentration were amplified to obtain two regression curves (one for the VIC—hulled—and one for the FAM—hulless) with the number of copies on the abscissa and the corresponding CTS (Cycle Threshold) in ordinate. In parallel to standard samples, analytical samples were amplified. The number of copies of the analytical samples was obtained by interpolation on the standard curves using the corresponding CTS. The software used is Sequence Detection Software 1.4.2-Applied Biosystems (Monza, Italy).

## 3. Results

### 3.1. Mono-Species Samples

The dPCR assay was evaluated for the specificity in wheat discrimination on the DNA extracted from the genotypes listed in Table 2. The DNA quantity extracted from such samples ranged from 19.5 to 42 ng/mg of sample, with a 1.80 mean ratio of absorbance at 260 nm and 280 nm, indicating an acceptable purity level.

**Figure 2:**
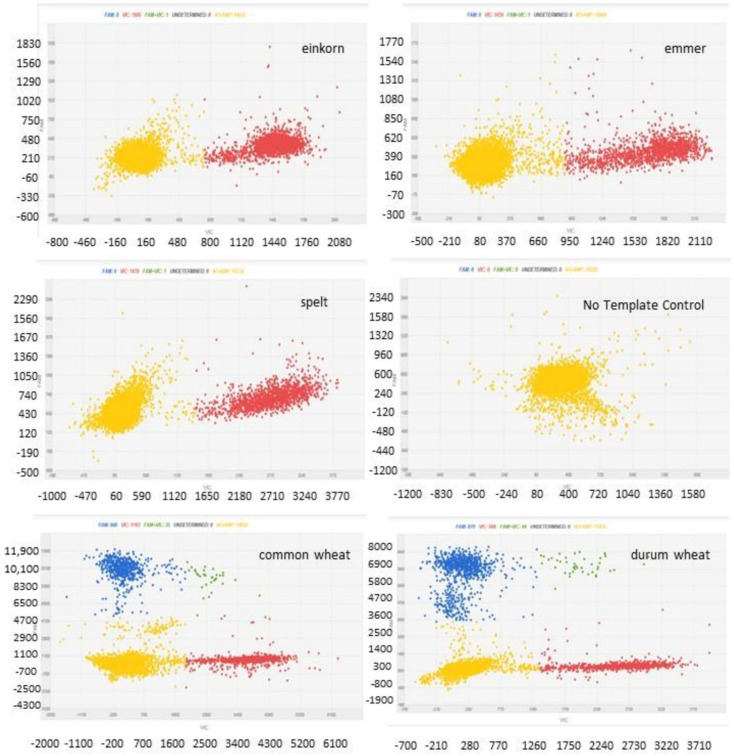
Two-dimensional scatter graphs generated by chip digital PCR (cdPCR) analysis of 100% hulled and hulless wheats. No template control is a blank sample without DNA, with negative partitions that contain no amplified targets (yellow signals). X axis stands for the VIC signal, whereas Y axis stands for the FAM signal. Einkorn, emmer and spelt carrying the recessive alleles show red (VIC) signal only, whereas hulless wheats, carrying both recessive and dominant alleles, show both red (VIC) and blue (FAM) signals. Green signals stand for partitions in which a co-amplification of both VIC and FAM targets occurs.

All the einkorn, emmer and spelt varieties showed VIC signal only, with an absent or negligible FAM signal (Figure 2). All the varieties showed FAM/VIC = 0, with a Pearson’s r correlation of 1 between experimentally and expected values. An FAM/VIC = 0, according to the calculation formulas of Figure 1, predicts 0% of durum or common wheats, as expected.

The durum and bread wheat varieties showed both VIC and FAM signals (Figure 2). The mean FAM/VIC ratio of common wheat samples was 0.52 ± 0.02 and those of durum wheat varieties was 0.95 ± 0.05, which are very close to the expected values of, respectively, 0.5 and 1. A Pearson’s r of 0.9989 was found between theoretical and experimentally obtained FAM/VIC ratios in hulless wheats. The FAM/VIC ratios experimentally obtained after analysis of nominal 100% common wheat and nominal 100% durum wheat samples were used to calculate the experimentally determined percentages using the polynomial curve of Figure 1. According to the calculation formulas of Figure 1, the mean, experimentally measured value for nominal 100% common wheat sample was 99.8 ± 0.44% and those for nominal 100% durum wheat sample was of 100%.

A subset of 18 DNA samples extracted from hulless and hulled wheats were analyzed independently by the CREA and CoopItalia laboratories and a Pearson’s r of 0.999 was found among the results obtained.

### 3.2. Mixed-Species Samples

The dPCR assay has been applied to the hulless wheat quantification in mixed-species samples prepared by mixing DNAs extracted from einkorn, emmer, spelt, durum and common wheat in the percentages reported in Table 4. The Pearson’s r between the expected and calculated hulless wheat percentages was 0.97. In the same Table 4 are reported the absolute and relative errors, as informative values about the precision and the accuracy of the method.

### 3.3. Commercial Samples

The dPCR assay has been applied to the panel of foods reported in Table 5, labeled as made from farro or containing farro among the ingredients. The products are commercially available and have been sampled on an Italian market. The products were chosen as representative of different food categories. A DNA quantity ranging from 1.5 to 17.5 ng/mg of sample has been obtained from commercial samples, with a 1.87 mean ratio of absorbance at 260 nm and 280 nm, indicating an acceptable purity level.

Table 5 shows the products and the cereal ingredients reported in the label. The percentages of farro experimentally determined with dPCR analysis by the two independent laboratories of CREA and CoopItalia are shown in the table. The data obtained by the CREA and CoopItalia laboratories are very close, with a Pearson’s r of 0.99. For several products (i.e., cookie 1, breakfast cereals, pearled farro, bread substitute 1, egg pasta, cookie 2 and baby food), the experimentally determined percentages fully confirm those reported in the labels, with a Pearson’s r of 0.99. The “Mix for bread making” sample showed a higher percentage of farro in comparison with those reported in the label, whereas the pasta sample contains farro as major ingredients, but even a percentage of hulless wheat.

### 3.4. Specificity

**Figure 3:**
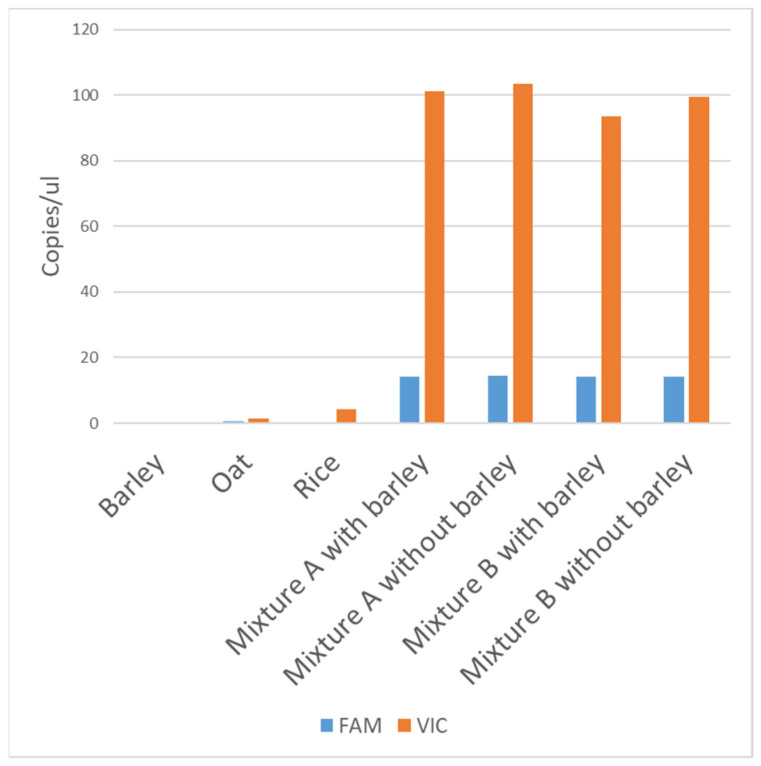
Copies/µL of FAM and of VIC targets. A total of 20 ng of barley, oat and rice DNA was amplified in dPCR. Mixture A was obtained by mixing 15 ng of hulled wheat DNA plus 4 ng of hulless wheat, with or without 1 ng of barley DNA. Mixture B was obtained by mixing 14 ng of hulled wheat DNA plus 4 ng of hulless wheat, with or without 2 ng of barley DNA.

The specificity of the assay has been evaluated considering cereals that can be present in the mixture of ingredients in a food sample. Barley, oat and rice fail to give amplification signals, or give very low signals, as reported in Figure 3. Moreover, mixtures of hulled and hulless wheats with or without barley have been evaluated, as reported in the same Figure 3. Very close FAM and VIC amplification signals were obtained for the mixtures with or without barley, suggesting that the presence of barley has no significant impact on the results of the analysis, supporting the assay specificity.

### 3.5. Real-Time PCR Assay

The same commercial samples reported in Table 5 have been even analyzed with the real-time qPCR assay (Table 6), based on the use of the same primers/probes of dPCR and of the same calculation approach reported in Section 2.2.3. In the qPCR analysis, a double standard curve has been generated, considering dilutions of hulled and hulless wheats.

Table 6 shows the percentages of farro experimentally determined with qPCR analysis. A Pearson’s r of 0.99 has been found between the hulled wheat percentages reported in the label and the values experimentally determined with qPCR analysis. The mean values obtained with dPCR and qPCR analyses on this set of commercial samples are very close, with a Pearson’s r of 0.98.

## 4. Discussion

A chip digital PCR assay has been developed for the discrimination between hulled and hulless wheats and their quantification along food chains. The assay is based on an allelic variation linked to the hulless/hulled seed morphology in wheats. Because einkorn, emmer and spelt seeds have the common characteristic to be hulled, whereas common and durum wheats are hulless, the dPCR assay can be used for the discrimination of the two wheat classes (i.e., hulless vs. hulled). The polymorphism targeted by the assay is localized in miRNA172 target site of the AP2-5 transcription factor on chromosome 5A that is involved in wheat threshing [26]. Polynomial curves have been developed starting from the nominal FAM/VIC ratios to calculate the hulled/hulless wheats percentages in experimental samples.

The assay has been evaluated on a panel of samples, including pure common and durum wheats, einkorn, emmer and spelt, hulless/hulled mixtures and commercial samples. From the results obtained, it can be concluded that the assay can be efficiently applied to the precise quantification of einkorn, emmer and spelt in mixture with common wheat or in mixture with durum wheat.

To evaluate the trueness of the method, defined as the degree of agreement of the expected value with the true value, the guidelines for GMO testing has been adopted. The main reason to adopt such guidelines lies in the fact that dPCR has so far been massively applied especially in the traceability of GMOs [29,30], which implies even the species traceability. The guidelines suggested for GMO can, therefore, likely be exploited for the traceability of *Triticum* species and subspecies. The same approach to evaluate trueness has been used by Köppel et al. [22] for the quantification of common wheat in spelt. According to such guidelines, the difference between the analytical value found compared to the certified, reference value must remain within the limits of 25% [31,32]. The trueness of our method fits the purpose: the estimated percentages were within the recommended ±25% acceptable bias.

The accuracy of the method decreases in samples in which, in addition to hulled wheats, blends of common and durum wheat are present in not-declared relative percentages. This situation, i.e., blends in unknown proportion of common and durum wheats together with hulled wheats, is probably not frequent. Durum wheat is in fact classically used in the pasta supply chain or the preparation of special types of bread. However, in such infrequent samples, the mean polynomial curve can be used for the percentage calculation of farro, as shown in Figure 1, with the awareness of obtaining an average quantification. The acceptability of this average quantification depends on the labeling requirements. Alternatively, it can be proposed to use two digitalPCR methods in series: the first, published by Morcia et al. [33], allows to quantify the percentage of hexaploid wheat compared to diploid wheat, while the second assay, proposed in this paper, provides the precise quantification of einkorn, emmer or spelt in samples containing common wheat, durum wheat or their mixtures at known percentages. Very recently, Köppel et al. [22] proposed an efficient dPCR assay designed on the Q-locus for the quantification of contaminations by common wheat in spelt-based products. However, in Italy and in other Mediterranean countries, emmer and durum wheat are the species of greater diffusion among, respectively, hulled and hulless wheats. Consequently, the dPCR assay developed in this study adds the ability to quantify hulless wheat contaminations in einkorn, emmer and spelt flours and derived food products. In addition to contamination by common wheat, our assay also takes into consideration that of durum wheat, a species of greater diffusion in Italy [34] and in other Mediterranean environments than common wheat. Moreover, the analytical problems due to the co-presence of common and durum wheat as contaminants are also introduced.

The primers/probes developed firstly for dPCR application have been additionally evaluated in a real-time PCR system. Two main reasons suggested to check the applicability of the assay with a real-time PCR instrument. The first reason is that the dPCR instruments are not, at the present time, as widespread as real-time ones, and therefore, there may be an interest, for laboratories that do not have the dPCR, to exploit the assay with a real-time PCR machine. The second reason is linked to the comparative evaluation of the analytical costs of the assay using the dPCR in comparison with a real-time one. Taking into account the reagents’ costs and the analytical time required, the cost of the dPCR approach in our hands is reduced by 30% compared to real-time PCR. The increased cost in real-time PCR is due to the higher amount of reagents required for each reaction and the need of standard curves’ development.

## 5. Conclusions

A new dPCR assay to quantify hulless wheat contamination in raw materials and premium food made of einkorn, emmer or spelt has been developed. The innovation in comparison with already available DNA-based methods is in the technique adopted, simpler and faster and able to do a precise quantification. This same technique has been exploited in the study of Köppel et al. [22] using a different polymorphism of the Q-locus and focusing mainly on the discrimination between common wheat and spelt. In our work, all hulled wheat species (i.e., einkorn, emmer and spelt) and all hulless (i.e., durum and common wheats) were considered. In conclusion, dPCR is confirmed as a particularly promising analytical method for the identification and quantification of plant species, in defense of the authenticity of the product. The potential of this technique has been understood by the food industry and retailers, which collaborate in the development and validation of these methods. Contrary to what was thought at the dawn of its diffusion, this technique allows considerable savings, both in terms of analytical times and reagents, as verified in our work. This aspect is also even more true by observing the recent advances in digital PCR instrumentation, which allow for flexibility and scalability of the analyses such as to further reduce analytical costs.

## Figures and Tables

**Table 1:** Analytical assays proposed for the identification and quantification of hulled wheats.

Analytical Target	Method	Reference
Spelt	Fatty acids profile	[14]
*Triticum* species	PCR-RFLP (*Q-locus*)	[15]
Spelt	RLP-LOC-CE, Real-time PCR (*γ-gliadin*)	[16]
Farro della Garfagnana in cereal mixtures	padlock probe ligation and multiplex microarray	[17]
Spelt	LC-MS peptide markers identification	[18]
Einkorn, emmer and spelt	tubulin-based polymorphism (TBP)	[19]
Spelt	PCR (*γ-gliadin*, *Q-locus*); NIR	[20]
Italian emmer landraces	Spectroscopy and chemometrics	[21]
Spelt	Duplex droplet digital PCR (*Q-locus*)	[22]

**Table 2:** Hulled and hulless wheat varieties used and their maintainers, defined as the natural or the legal person identified by the national seed law as responsible for maintaining the variety in purity, very often coincident with the breeder [27]. A = Fondazione M. Bolognini, Sant’Angelo Lodigiano (Lodi), Italy; B = CREA—Consiglio per la Ricerca in Agricoltura e l’Analisi dell’Economia Agraria, Italy; C = Prometeo s.r.l., Urbino (PU), Italy; D = Istituto Di Genetica Vegetale CNR, Bari, Italy; E = Agribosco s.r.l., Sigillo (PG), Italy; F = Società Produttori Sementi, Bologna, Italy; G = Società Italiana Sementi, San Lazzaro di Savena (BO), Italy.

Botanical Species	Variety	Maintainers
*Triticum monococcum* L.	Antenato	A,B
*Triticum monococcum* L.	Hammurabi	A,B
*Triticum monococcum* L.	Monili	A,B
*Triticum monococcum* L.	Monlis	B,C
*Triticum monococcum* L.	Norberto	A,B
*Triticum dicoccum* Schubler	Augeo	E
*Triticum dicoccum* Schubler	Farvento	D
*Triticum dicoccum* Schubler	Giovanni Paolo	B
*Triticum dicoccum* Schubler	Hervillum	E
*Triticum dicoccum* Schubler	Padre Pio	B
*Triticum dicoccum* Schubler	Rosso Rubino	C
*Triticum dicoccum* Schubler	Yakub	C
*Triticum dicoccum* Schubler	Zefiro	C
*Triticum dicoccum* Schubler	Sephora	-
*Triticum spelta* L.	Benedetto	A,B
*Triticum spelta* L.	Forenza	D
*Triticum spelta* L.	Giuseppe	A,B
*Triticum spelta* L.	Maddalena	B
*Triticum spelta* L.	Pietro	A,B
*Triticum spelta* L.	Rita	B
*Triticum spelta* L.	Rossella	B
*Triticum durum*	Aureo	F
*Triticum durum*	Iride	F
*Triticum durum*	Cappelli	B,G
*Triticum aestivum*	Apulia	B
*Triticum aestivum*	Bologna	G
*Triticum aestivum*	Palesio	G

**Table 3:** Sequence variation at the miR172 target site in different homoeologs and in wheat species different in ploidy level and grain threshability.

Wheat Species and Threshing Habit	Chromosome 5A miR172 Target Site	Chromosome 5B miR172 Target Site	Chromosome 5D miR172 Target Site
Einkorn Non-free-threshing (GenBank MK101270.1)	gct gca gca tca tca gga tt**c** tct	-	-
Emmer Non-free-threshing (GenBank MK493450.1)	gct gca gca tca tca gga tt**c** tct	gct gca gca tca tca gga tt**c** tct	-
Spelt Non-free-threshing (GenBank MK450625.1)	gct gca gca tca tca gga tt**c** tct	gct gca gca tca tca gga tt**c** tct	gct gca gca tca tca gga tt**c** tct
Durum wheat Free-threshing (GenBank KY924305.1)	gct gca gca tca tca gga tt**t** tct	gct gca gca tca tca gga tt**c** tct	-
Common wheat Free-threshing (GenBank JF701619.1)	gct gca gca tca tca gga tt**t** tct	gct gca gca tca tca gga tt**c** tct	gct gca gca tca tca gga tt**c** tct

**Table 4:** Actual hulless wheat percentages in comparison with those experimentally determined in samples obtained by mixing DNA extracted from pure einkorn, emmer, spelt, common and durum wheats.

Nominal Hulled to Hulless Ratio in Mixed Samples	Measured Hulless Wheat Percentage	Absolute Error	Relative Error
80% einkorn, 20% durum wheat	20%	0	-
80% emmer, 20% durum wheat	25%	5	0.20
80% spelt, 20% durum wheat	24%	4	0.16
50% einkorn, 50% durum wheat	50%	0	-
50% emmer, 50% durum wheat	55%	5	0.09
50% spelt, 50% durum wheat	50%	0	-
40% einkorn, 60% durum wheat	55%	5	0.09
40% emmer, 60% durum wheat	69%	9	0.13
40% spelt, 60% durum wheat	64%	4	0.06
50% einkorn, 50% common wheat	48%	2	0.04
50% emmer, 50% common wheat	52%	2	0.04
50% spelt, 50% common wheat	54%	4	0.07

**Table 5:** Commercially available food sampled, their cereal content as reported in the label and hulled wheat percentages determined by the two CREA and CoopItalia laboratories using dPCR assay.

Commercial Sample	Cereal Formulation in the Label	Farro % (dPCR Determined by CREA Lab)	Farro % (dPCR Determined by CoopItalia Lab)
Cookie 1	Farro 54%, common wheat	54%	56%
Breakfast cereals	Whole farro flakes 100%	100%	97%
Pearled farro	Farro 100%	100%	100%
Bread substitute 1	Farro 99.8%	99%	99%
Mix for bread making	Whole farro flour 7%, common wheat flour 93%	25%	25%
Flour	Spelt flour	90%	91%
Bread substitute 2	Common wheat flour, common wheat flakes 5.1%, toasted wheat bran, whole farro flour 2.1%, malted common wheat flour	7%	0%
Pasta	Farro flour	78%	76%
Egg pasta	Farro flour 80.64%	81%	83%
Bread substitute 3	Common wheat flour, farro flour 30.4%, malt, oat flakes	25%	25%
Cookie 2	Farro flour	100%	100%
Baby food	Farro 100%	100%	100%

**Table 6:** Commercially available food sampled, their cereal content as reported in the label and hulled wheat percentages determined using a qPCR assay.

Commercial Sample	Cereal Formulation in the Label	Farro % qPCR Determined
Cookie 1	Farro 54%, common wheat	58%
Breakfast cereals	Whole farro flakes 100%	100%
Pearled farro	Farro 100%	100%
Bread substitute 1	Farro 99.8%	100%
Mix for bread making	Whole farro flour 7%, common wheat flour 93%	0%
Flour	Spelt flour	95%
Bread substitute 2	Common wheat flour, common wheat flakes 5.1%, toasted wheat bran, whole farro flour 2.1%, malted common wheat flour	0%
Pasta	Farro flour	90%
Egg pasta	Farro flour 80.64%	94%
Bread substitute 3	Common wheat flour, farro flour 30.4%, malt, oat flakes	27%
Cookie 2	Farro flour	100%
Baby food	Farro 100%	100%

## Data Availability

Not applicable.
